# Brain Insulin Signaling is Associated with Late-Life Cognitive Decline

**DOI:** 10.14336/AD.2023.1117

**Published:** 2024-10-01

**Authors:** Han Tong, Ana W. Capuano, Owen T. Carmichael, Kathryn L. Gwizdala, David A. Bennett, Rexford S. Ahima, Steven E. Arnold, Zoe Arvanitakis

**Affiliations:** ^1^Rush Alzheimer’s Disease Center, Rush University Medical Center, Chicago, IL, USA.; ^2^Pennington Biomedical Research Center, Baton Rouge, LA, USA.; ^3^Division of Endocrinology, Diabetes, & Metabolism, Department of Medicine, Johns Hopkins University School of Medicine, Baltimore, MD, USA.; ^4^Alzheimer's Clinical and Translational Research Unit, Department of Neurology, Massachusetts General Hospital, Harvard Medical School, Boston, MA, USA.

**Keywords:** insulin signaling, cognitive decline, brain, postmortem, AKT

## Abstract

Type-2 diabetes is associated with an increased risk of dementia, and the underlying mechanism might involve abnormal insulin signaling in the brain. The objective of this study was to examine the association of postmortem brain insulin signaling with late-life cognitive decline. Among participants of Religious Orders Study, a community-based clinical-pathological cohort, 150 deceased and autopsied older individuals (75 with diabetes matched to 75 without by age at death, sex, and education) had postmortem brain insulin signaling measurements collected in the prefrontal cortex using ELISA and immunohistochemistry. By using adjusted linear mixed-effects models, we examined the association of postmortem brain insulin signaling with late-life cognitive function assessed longitudinally (mean follow-up duration = 9.4 years) using a battery of neuropsychological tests. We found that a higher level of serine/threonine-protein kinase (AKT) phosphorylation (pT^308^AKT1/total AKT1) was associated with a faster decline in global cognition (estimate = -0.023, p = 0.030), and three domains: episodic memory (estimate = -0.024, p = 0.032), working memory (estimate = -0.018, p = 0.012), and visuospatial abilities (estimate = -0.013, p = 0.027). The level of insulin receptor substrate-1 (IRS1) phosphorylation (pS^307^IRS1/total IRS1) was not associated with decline in global cognition or most cognitive domains, except for perceptual speed (estimate = 0.020, p = 0.020). The density of pS^616^IRS1-stained cells was not associated with decline in global cognition or any of the domains. In conclusion, these findings provide novel evidence for an association between brain insulin signaling and late-life cognitive decline. AKT phosphorylation is associated with a decline in global cognition and memory in particular, whereas IRS1 phosphorylation is associated with a decline in perceptual speed.

## INTRODUCTION

Type-2 diabetes mellitus (T2DM) and cognitive impairment are two of the most prominent and disabling chronic conditions of aging that negatively impact older individuals and their families. It is estimated that 29.2% of older adults aged over 65 in the US have T2DM (National Diabetes Statistics Report, Centers for Disease Control and Prevention), whereas approximately 32% have either mild cognitive impairment or dementia [1]. Accumulating evidence suggests that T2DM and cognitive impairment are linked [2-4]. Notably, a recently published large cohort study with more than 30 years of follow-up found that, compared with participants without T2DM at 65, those with T2DM onset 6 to 10 years earlier had a doubled risk of subsequent dementia [5]. However, the precise mechanisms by which older people with T2DM become susceptible to cognitive impairment remain unclear.

Given that a major aspect of the pathophysiology of T2DM is insulin resistance [6], and that insulin receptors are densely expressed in the brain [7], one possible pathway by which T2DM increases the risk for cognitive impairment may be via insulin resistance in the brain. By examining postmortem brain tissues, previous studies have consistently found insulin signaling abnormalities in patients with Alzheimer’s disease (AD), a leading cause of dementia in older adults [8-12]. We previously reported that neurons in the hippocampal formation of patients with AD exhibited marked abnormalities in the phosphorylation states of key protein mediators of the canonical insulin signaling pathway, which involves the sequential activation of insulin receptor (IR), insulin receptor substrate 1 (IRS1), phosphoinositide 3-kinase (PI3K), and alpha serine/threonine-protein kinase (AKT1) [13]. Hyper-phosphorylated IRS1 and downstream kinases such as AKT1 were not only associated with increased levels of AD pathology but also associated with more memory loss proximate to death in older adults[13]. AKT1 links this pathway to the signature pathologies of AD. Activated AKT1 inhibits glycogen synthesis kinase 3 (GSK3β), a constitutively active kinase promoting tau phosphorylation and also regulating the expression of β-site amyloid precursor protein cleaving enzyme 1 (BACE1) and insulin-degrading enzyme (IDE) [14]. Thus, abnormal interactions between AKT1 and GSK3β may promote the accumulation of paired-helical filament tau and amyloid-β plaque pathologies [14]. Sustained AKT1 activation also indirectly activates mammalian target of rapamycin complex 1 (mTORC1), which inhibits IRS1 and further contributes to insulin resistance in a vicious cycle [15, 16]. In a recent study, we showed that higher levels of AKT1 phosphorylation (pT^308^AKT1/total AKT1) were associated with higher levels of global AD neuropathology and lower levels of global cognitive function, working memory and episodic memory proximate to death in community-based older individuals [17]. However, our prior study did not address whether brain insulin signaling is related to changes in cognitive function over time, a more clinically meaningful health outcome.

Building on our previous work, we sought to examine the association between brain insulin signaling and late-life cognitive decline. We measured the levels of insulin signaling molecules (IRS1, AKT1) in the prefrontal cortex of 150 older adults (75 with, and 75 without diabetes) using enzyme-linked immunoassay (ELISA) and immunohistochemistry. By using adjusted mixed-effects regression analyses, we examined the associations of brain insulin signaling measures with longitudinally assessed cognitive function. In additional analyses, we examined the effects of diabetes status and the presence of *APOEε4* allele.

## MATERIALS AND METHODS

### Participants, Clinical Evaluations, and Selection of Cases

All participants in this study were from the Religious Orders Study (ROS), a prospective, community-based, clinical-pathologic cohort study of aging, which is ongoing [18]. Approved by the Institutional Review Board of Rush University Medical Center, ROS began enrolling Catholic nuns, priests, and brothers in 1994 from convents and monasteries across the United States. Before being enrolled, each study participant signed an informed consent form to undergo yearly testing and an anatomical gift act to donate the brain at the time of death, and a repository consent to share resources.

The ROS participants underwent annual clinical evaluations including medical history, physical exam and neuropsychological testing. Nineteen cognitive tests were administered, of which 17 were grouped to form composite measures of global cognition and five cognitive domains including episodic memory, working memory, semantic memory, perceptual speed, and visuospatial abilities [19]. Using the baseline mean and standard deviation from the entire cohort, we converted the raw scores of individual tests to z scores. These z-scores were averaged across tests to create composite scores for global cognition or individual cognitive domains. The presence of diabetes was determined based on medical history, visually inspected antidiabetic medications or a combination of both [20]. Data on depressive symptoms (based on the 10-item Center for Epidemiological Studies Depression Scale) and the presence of the Apolipoprotein E ε4 (*APOEε4*) allele were also collected as previously described [18].

Of 1194 people who enrolled at the time of analyses, 669 died and 622 came to autopsy. After a series of pre-determined exclusion criteria were applied, as described in detail previously [17], a total of 150 participants, 75 with and 75 without diabetes were matched by sex on continuous measures of age at death and education, using a propensity score-based algorithm, and included for subsequent analyses. The demographic, cognitive, and neuropathological characteristics of these 150 participants are reported in the Results section. Briefly, the mean age at death was 86.6 ± 6.1 years, and 48% of study participants were female, as previously published [17]. On average, participants had 18.1 ± 3.3 years of education.

### Brain Autopsy and Insulin Signaling Measures

All brain autopsies were performed following a standardized procedure, with tissues from one hemisphere being frozen to -80°C and from the other, being fixed with paraformaldehyde [18]. The autopsy rate exceeded 90%, and the mean postmortem interval was 9.6 hours [21].

Brain insulin signaling measures were quantified in the dorsolateral prefrontal cortex within the middle frontal gyrus cortex (MFC), as previously described [17]. Previously frozen brain samples (250 mg) were thawed and homogenized in lysis buffer containing protease and phosphatase inhibitors. Supernatant aliquots containing 50 pg protein were assayed in duplicate along with protein standards in microtiter plate wells using PathScan enzyme-linked immunosorbent assay (ELISA) kits (Cell Signaling Technology, Danvers, MA), via solid-phase sandwich ELISA method. The quantified brain insulin signaling proteins and the catalog numbers of the ELISA kits we used were as follows: total IRS1, #7328; pIRS1 (S307), #7287; AKT1, #7170; pAKT1 (T308), #7252. After incubation with lysate, the protein was captured by the coated antibody. Following extensive washing, a specific antibody was added to detect the captured protein and HRP substrate, tetramethylbenzidine, was added to develop color. The absorbance was read at 450nm on Epoch Microplate Spectrophotometer (Agilent Technologies, formerly BioTek Instruments, Winooski, VT). Absorbance for the developed color is proportional to the quantity of protein. For analyses, the pS^307^IRS1/total IRS1 and pT^308^AKT1/total AKT1 ratios were calculated to estimate IRS1 phosphorylation and AKT1 phosphorylation, respectively.

In addition, immunohistochemistry for pS^616^IRS1 was conducted in 10μm-thick tissue sections from paraformaldehyde-fixed, paraffin-embedded MFC blocks, as previously described [17]. Briefly, the tissue sections were dewaxed in xylenes, rehydrated in descending ethanols, quenched of endogenous peroxidase activity in 5% H_2_O_2_, boiled in 1 mM ethylenediaminetetraacetic acid for epitope retrieval, rinsed in buffer, and blocked in 10% normal horse serum. Primary antibody against pS^616^IRS (#44-550G, Thermo Fisher Scientific, Waltham, MA; rabbit 1:500) was used for incubation of pretreated tissues overnight at 4°C. Next, sections were incubated in HRP-conjugated goat anti-rabbit secondary antibody (#ALI4404, Thermo Fisher Scientific, Waltham, MA) followed by avidin-biotin-peroxidase complex for 1 hour, and finally reacted with a 0.05% diaminobenzidine (DAB)-0.03% hydrogen peroxide solution for 10 minutes. The immunoreaction signal was enhanced by adding NiSO4 (0.25% final dilution) to the DAB solution. The primary antibody directed at pS^616^IRS1 and secondary reagents in this study had been validated and used as described in previous publications [12, 13, 17]. Antibody specificity of the pS^616^IRS1 antibody had been demonstrated with Western blotting per manufacturer, and negative control slides without primary antibody were included showing absence of staining and low background. We used a Glissando Slide Scanner (Objective Imaging, Cambridge, UK) for automated microscopy digital image capture (contiguous montage of 200x magnification grayscale photo-micrographs covering the full depth of MFC region of interest present on the tissue section) and Image-Pro Plus software (Media Cybernetics, Rockville, MD) for computer-assisted image analysis to obtain the number of pS^616^ IRS1 positive neuronal cytoplasmic profiles per mm^2^ for analyses within portions of MFC where the pial surface and gray-white matter border were parallel, avoiding sulcal or gyral flexures where cell densities either compress or expand. Representative photomicrographs of immunohistochemical labeling of MFC sections for pS^616^IRS1 have been published elsewhere [17].

### Statistical Approach

The initial analysis included a detailed examination of data distribution and the correlation structure of the brain insulin signaling variables of interest. There were two categories of brain insulin signaling variables. The first category was ELISA, with 2 main variables: pS^307^IRS1/total IRS1 ratio and pT^308^AKT1/total AKT1 ratio. The second was immunohistochemistry, with 1 variable: pS^616^IRS1 cells/mm^2^. To examine the associations between brain insulin signaling and longitudinally assessed late-life cognitive function, we started by fitting separate linear mixed-effects regression models with cognition as the outcome variable and each of the brain insulin signaling measures as the predictor variable including participants with two or more observations (n=147). Given that late-life cognitive decline also has a non-linear component resulting a gradual accelerating course of decline close to death [22], we complemented the linear mixed-effects regression analyses with a nonlinear, sigmoidal mixed-effects model developed by our group [23]. This regression model is based on four parameters: the initial level of cognition, the final level of cognition at death, the midpoint of cognitive decline (the point in time at which half of the total decline occurred from baseline until death), and the nonlinear rate of cognitive decline (the nonlinear trajectory between the initial and final cognitive levels) [24]. Lastly, we explored whether diabetes status and the presence *APOEε4* allele affect the association between brain insulin signaling measures and cognitive decline in separate linear mixed-effects regression models. All models were adjusted for age at death, sex, and education. Age and education were centered on their means for interpretation purposes. A two-tailed hypothesis was assumed. The linear mixed-effects regression analyses were conducted using SAS/STAT software, version 9.4 of the SAS system for Linux (SAS Institute, Cary, NC). The nonlinear sigmoidal analyses were conducted using the “nlive” package [25] in R software, version 4.2.2 (R Foundation for Statistical Computing, Vienna, Austria).

## RESULTS

### Sample Characteristics and Brain Insulin Signaling Measures

The demographic, cognitive, and neuropathologic characteristics of the 150 study participants have been reported previously [17]. We did not find significant difference between participants with diabetes and those without diabetes in demographic characteristics (age at death, sex, and education), in the level of cognitive function measured within one year before death, in last-valid or study-average depressive symptoms, and in the severity of postmortem AD pathology (all p>0.05). There were 147 persons with longitudinal cognitive data, and the mean duration of follow-up was 9.4 ± 4.3 years.

The median values of brain insulin signaling measures in participants with diabetes were consistently higher than those in participants without diabetes. However, we only found significant pairwise difference in pS^307^IRS1/total IRS1 (mean difference: 0.05, 95% confidence interval: 0.01-0.09), as reported in our previous work [17].

**Table 1:** Associations between brain insulin signaling measures and the decline of late-life cognitive function (N=147).

Predictors	Estimate (SE, p-value)					
	Global Cognition	Episodic Memory	Working Memory	Semantic Memory	Perceptual Speed	Visuospatial Abilities
ELISA						
pS^307^IRS1/total IRS1						
Main Effect	0.082 (0.091,0.373)	0.062 (0.112,0.584)	0.003 (0.070,0.970)	0.186 (0.100,0.066)	0.233 (0.087,0.008)	0.080 (0.069,0.252)
Interaction with Time	0.009 (0.011,0.396)	0.011 (0.012,0.337)	0.006 (0.007,0.419)	0.0153 (0.011,0.171)	0.020 (0.009,0.020)	0.007 (0.006,0.248)
pT^308^AKT1/total AKT1						
Main Effect	-0.251 (0.088,0.005)	-0.344 (0.108,0.002)	-0.203 (0.067,0.003)	-0.144 (0.100,0.151)	-0.129 (0.087,0.142)	-0.105 (0.067,0.117)
Interaction with Time	-0.023 (0.010,0.030)	-0.024 (0.011,0.032)	-0.018 (0.007,0.012)	-0.008 (0.011,0.460)	-0.012 (0.009,0.177)	-0.013 (0.006,0.027)
Immunohistochemistry						
pS^616^IRS1 cells/mm^2^						
Main Effect	-0.073 (0.086,0.397)	-0.087 (0.105,0.410)	-0.013 (0.067,0.848)	-0.132 (0.096,0.170)	-0.062 (0.084,0.465)	-0.086 (0.066,0.193)
Interaction with Time	0.002 (0.011,0.840)	-0.002 (0.012,0.850)	0.003 (0.008,0.696)	-0.003 (0.011,0.799)	0.005 (0.009,0.614)	-0.002 (0.006,0.722)

Note: All linear mixed-effects regression models were adjusted for age at death, sex, education, and their interaction with time. Data were from 147 participants with at least two longitudinal observations of cognitive function. Bold values denote statistical significance (p<0.05).

### Brain Insulin Signaling and Global Cognitive Function

Using linear mixed-effects regression models adjusting for age at death, sex, education, and their interaction with time, we first examined the associations between brain insulin signaling measures and a decline in global cognitive function over the course of the study. As shown in Table 1, we found that a higher level of AKT1 phosphorylation (pT^308^AKT1/total AKT1) was associated with both a lower level of global cognitive function proximate to death (main effect of the predictor) and a faster rate of cognitive decline (interaction of the predictor with time). There was no association between any of the other brain insulin signaling measures, including the level of IRS1 phosphorylation (pS^307^IRS1/total IRS1) and the density of pS^616^IRS1 cells (pS^616^IRS1 cells/mm^2^), with the level or with change in global cognition.

**Figure 1.**
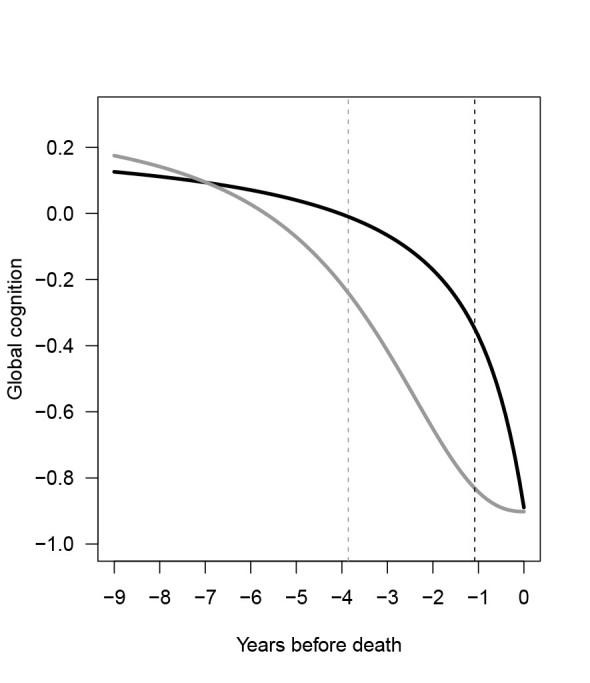
The non-linear trajectories of late-life global cognition, by two pT^308^AKT1/total AKT1 levels. The curves show the trajectories of global cognition during the last nine years of life by two pT^308^AKT1/total AKT1 levels. The black curve represents the trajectory for study participants with pT^308^AKT1/total AKT1 level at 25^th^ percentile, and the gray curve shows the trajectory for participants at 75^th^ percentile, based on a sigmoidal mixed-effects model adjusting for age at death, sex, and education (N=147). The black and gray dash lines show the point in time at which half of the cognitive decline occurred for participants with pT^308^AKT1/total AKT1 level at 25th percentile and 75th percentile, respectively.

Since cognitive decline close to death is non-linear, we examined the association between brain insulin signaling measures and the decline in global cognition using the sigmoidal mixed-effects model. We found that the midpoint of decline in global cognition occurred earlier with higher levels of AKT1 phosphorylation (estimate = 6.484, standard error = 0.448, p < 0.001). The nonlinear rate of decline in global cognition was also influenced by the level of AKT1 phosphorylation (estimate = 1.258, standard error = 0.214, p < 0.001), indicating an association with the non-linearity of the decline in global cognition. These effects are illustrated in Figure 1, which shows cognitive trajectories for participants with a pT^308^AKT1/total AKT1 level at the 25^th^ percentile and the 75^th^ percentile.

### Brain Insulin Signaling and Cognitive Domains

Next, we conducted secondary analyses using similarly adjusted mixed-effects regression models to examine the associations between brain insulin signaling measures and cognitive decline within each of the five separate domains (i.e., episodic memory, working memory, semantic memory, perceptual speed, and visuospatial abilities). We found that a higher level of AKT1 phosphorylation (pT^308^AKT1/total AKT1) was associated with a lower level of episodic memory and working memory proximate to death, and a faster rate of decline in episodic memory, working memory, and visuospatial abilities. In addition, we found that a lower level of IRS1 phosphorylation (pS^307^IRS1/total IRS1) was associated with a lower level of perceptual speed proximate to death, and a faster rate of its decline. There was no other association of the insulin signaling measures with the level or change in cognitive domains.

**Table 2:** The associations of diabetes status and its interaction with brain insulin signaling measures with the decline of late-life cognitive function (N=147).

Predictors	Estimate (SE, p-value)					
	Global Cognition	Episodic Memory	Working Memory	Semantic Memory	Perceptual Speed	Visuospatial Abilities
Diabetes						
Main Effect	-0.098 (0.182,0.592)	-0.144 (0.225,0.522)	-0.046 (0.138,0.741)	-0.017 (0.202,0.932)	-0.173 (0.176,0.327)	-0.050 (0.136,0.715)
Interaction with Time	-0.002 (0.021,0.924)	-0.003 (0.023,0.888)	-0.006 (0.014,0.674)	0.007 (0.022,0.744)	-0.018 (0.017,0.305)	-0.009 (0.011,0.417)
pS^307^IRS1/total IRS1*Diabetes						
Main Effect	-0.005 (0.178,0.979)	-0.076 (0.219,0.731)	-0.009 (0.136,0.950)	0.040 (0.196,0.838)	0.038 (0.168,0.820)	0.062 (0.135,0.646)
Interaction with Time	-0.002 (0.021,0.940)	-0.014 (0.023,0.534)	0.011 (0.014,0.442)	-0.002 (0.022,0.932)	0.003 (0.017,0.853)	0.009 (0.012,0.465)
pT^308^AKT1/total AKT1*Diabetes						
Main Effect	-0.206 (0.177,0.246)	-0.330 (0.215,0.127)	-0.106 (0.134,0.433)	-0.176 (0.200,0.382)	0.011 (0.175,0.952)	-0.034 (0.134,0.801)
Interaction with Time	-0.015 (0.021,0.473)	-0.030 (0.023,0.192)	0.005 (0.015,0.718)	-0.013 (0.022,0.558)	-0.005 (0.017,0.777)	-0.011 (0.011,0.327)
pS^616^IRS1 cells/mm^2^*Diabetes						
Main Effect	-0.252 (0.175,0.153)	-0.343 (0.213,0.110)	-0.075 (0.137,0.585)	-0.395 (0.193,0.043)	0.070 (0.171,0.683)	-0.166 (0.133,0.216)
Interaction with Time	-0.026 (0.022,0.240)	-0.024 (0.024,0.323)	-0.026 (0.017,0.117)	-0.046 (0.023,0.051)	0.014 (0.019,0.471)	0.008 (0.014,0.593)

Note: All linear mixed-effects regression models were adjusted for age at death, sex, education, and their interaction with time. Data were from 147 participants with at least two longitudinal observations of cognition. Bold values denote statistical significance (p<0.05).

### Brain Insulin Signaling, Cognitive Function, and Diabetes

Given that the link between brain insulin signaling and diabetes remains unclear, we also tested whether diabetes was related to cognition in this small sample of persons with and without diabetes. We did not find significant associations of diabetes status with the level or the rate of decline in cognition (Table 2), which was likely due to a lack of statistical power. However, diabetes status interacted with the density of pS^616^IRS1-stained cells in associating with semantic memory (Table 2). A further analysis stratified by diabetes status (data not presented in the table) showed that, in those participants with diabetes, higher densities of pS^616^IRS1 stained cells were associated with lower levels of semantic memory (estimate = -0.324, standard error = 0.125, p = 0.012). In those without diabetes, densities of pS^616^IRS1 stained cells were not associated with the level semantic memory (estimate = 0.098, standard error = 0.153, p = 0.522). Diabetes status did not change other associations between brain insulin signaling measures and cognition (Table 2).

### Brain Insulin Signaling, Cognitive Function, and APOEε4

*APOEε4* data were available in a subset of 116/147 (79%) participants with longitudinal cognitive data. The presence of one or more *APOEε4* allele was associated with both a lower level of global cognition (p = 0.005), as well as a faster rate of decline in global cognition (p = 0.003). In secondary analyses, we also found that *APOEε4* was associated with both a lower level and a faster rate of decline in episodic memory, working memory, semantic memory, and perceptual speed, but not visuospatial abilities (Table 3). The presence of *APOEε4* allele did not change any associations between brain insulin signaling measures and cognition (Table 3).

Given the association between *APOEε4* and cognition, we added terms for *APOEε4* and its interaction with time to the core linear mixed-effects regression models (Table 4). After adjusting for *APOEε4* (data available only in N=116), a higher level of pT^308^AKT1/total AKT1 remained associated with a lower level of global cognition, as well as with episodic memory and working memory, proximate to death. In addition, higher pT^308^AKT1/total AKT1 remained associated with a faster rate of decline in working memory and visuospatial abilities. However, the associations between pT^308^AKT1/total AKT1 and decline in global cognition and episodic memory were no longer significant after adjusting for *APOEε4*. Furthermore, the associations between pS^307^IRS1/total IRS1 and the level and change in perceptual speed were no longer significant.

When we removed *APOEε4* and its interaction with time from these models (N=116), the results (data not shown) were largely the same as those of *APOEε4*-adjusted regression models (as shown in Table 4), suggesting *APOEε4* did not significantly affect the association between brain insulin signaling measures and decline of cognitive function. The apparent “lost” associations were likely due to a loss of statistical power when using a smaller sample.

## DISCUSSION

In this community-based clinical-pathologic cohort study, we found associations between brain insulin signaling and late-life cognitive decline. In older adults with and without diabetes, higher levels of serine/threonine-protein kinase (AKT1) phosphorylation (pT^308^AKT1/total AKT1) in the brain were associated with lower levels and faster decline in global cognition. In addition, greater AKT1 phosphorylation was associated with lower levels in two cognitive domains (episodic memory and working memory) and faster decline in episodic memory, working memory, and visuospatial abilities. Brain insulin receptor substrate-1 (IRS1) phosphorylation (pS^307^IRS1/total IRS1) was not associated with the level or the rate of decline in global cognition or most cognitive domains, except for perceptual speed. These associations were present regardless of the presence of diabetes. The number of pS^616^IRS1 cells per mm^2^ in the brain was not associated with the level or rate of decline in global cognition or cognitive domains. Furthermore, by using a nonlinear regression model, we also demonstrated that a higher pT^308^AKT1/total AKT1 level was associated with an earlier nonlinear decline in global cognition. Taken together, these findings suggest that brain insulin signaling is associated with late-life cognitive decline and that AKT1 phosphorylation may be preferentially associated with a decline in memory, whereas IRS1 phosphorylation may be preferentially associated with perceptual speed.

To date, only a few studies have examined the association of brain insulin signaling with cognition, and all of them are cross-sectional. An earlier study of postmortem human tissue by our group, found that higher levels of serine-phosphorylated IRS1 (pS^616^IRS1 and pS6³6/6³? IRS1) in hippocampal formation were associated with lower cognitive function in older individuals, particularly in episodic memory and working memory domains [13].

**Table 3:** The associations of APOEε4 status and its interaction with brain insulin signaling measures with the decline of late-life cognitive function (N=147).

Predictors	Estimate (SE, p-value)					
	Global Cognition	Episodic Memory	Working Memory	Semantic Memory	Perceptual Speed	Visuospatial Abilities
*APOEε4*						
Main Effect	-0.657 (0.230,0.005)	-0.784 (0.284,0.007)	-0.469 (0.177,0.009)	-0.568 (0.269,0.037)	-0.584 (0.230,0.012)	-0.240 (0.181,0.187)
Interaction with Time	-0.075 (0.025,0.003)	-0.078 (0.029,0.006)	-0.039 (0.018,0.029)	-0.081 (0.029,0.006)	-0.071 (0.024,0.003)	-0.009 (0.015,0.563)
pS^307^IRS1/total IRS1 * *APOEε4*						
Main Effect	-0.132 (0.248,0.597)	-0.088 (0.307,0.775)	-0.141 (0.191,0.464)	-0.156 (0.291,0.592)	0.061 (0.250,0.808)	0.212 (0.518,0.684)
Interaction with Time	-0.015 (0.027,0.588)	-0.010 (0.032,0.758)	-0.020 (0.020,0.328)	-0.004 (0.032,0.890)	0.004 (0.026,0.882)	0.0558 (0.051,0.271)
pT^308^AKT1/total AKT1 * *APOEε4*						
Main Effect	0.114 (0.196,0.563)	0.150 (0.240,0.533)	0.069 (0.150,0.646)	0.156 (0.235,0.508)	0.060 (0.199,0.764)	-0.045 (0.156,0.773)
Interaction with Time	0.011 (0.022,0.625)	0.010 (0.025,0.690)	0.007 (0.016,0.657)	0.026 (0.026,0.312)	-0.005 (0.021,0.796)	-0.003 (0.014,0.812)
pS^616^IRS1 cells/mm^2^ * *APOEε4*						
Main Effect	0.470 (0.517,0.365)	0.707 (0.637,0.270)	0.143 (0.399,0.722)	0.545 (0.602,0.367)	0.212 (0.518,0.684)	0.357 (0.403,0.378)
Interaction with Time	0.056 (0.056,0.315)	0.096 (0.062,0.126)	-0.018 (0.038,0.639)	0.046 (0.065,0.480)	0.056 (0.051,0.271)	0.004 (0.031,0.907)

Note: All linear mixed-effects regression models were adjusted for age at death, sex, education, and their interaction with time. Data were from 147 participants with at least two longitudinal observations of cognition. Bold values denote statistical significance (p<0.05).

In a more recent community-based, clinical-pathologic study leveraging complementary assessment methods, we assessed the association between insulin signaling in the lateral prefrontal cortex and the level of cognitive function proximate to death[17]. In linear regression analyses, we did not find an association of brain IRS1 phosphorylation at the S^307^ site, with global cognition or any of the 5 cognitive domains proximate to death. However, we found that increased brain AKT1 phosphorylation at the T^308^ (pT^308^AKT1/total AKT1), a critical node downstream to IRS1 in insulin signaling, was associated with a lower level of global cognition proximate to death and with two of the five cognitive domains, specifically episodic memory and working memory [17]. Expanding on our previously published cross-sectional analyses, we conducted linear mixed-effects regression analyses to examine the association of postmortem brain insulin signaling with longitudinally assessed cognitive function in older adults. Our results not only confirmed the associations of pT^308^AKT1/total AKT1 with the level of global cognition and the two cognition domains (episodic memory and working memory), but also showed that higher AKT1 phosphorylation at T^308^ was associated with a faster rate of decline in global cognition, episodic memory, working memory, and visuospatial abilities. These longitudinal analyses provide enhanced confidence in the findings. Further, our results offer new insights into the more clinically relevant outcome of change in cognition, which is often of particular interest to clinicians and patients alike, given the more meaningful implications for future health and quality of life. Moreover, our longitudinal analyses provide new information that lower IRS1 phosphorylation at S^307^ is associated with a lower level and faster rate of decline in perceptual speed, but not in global cognition and other cognitive domains. As a whole, insulin signaling appears to be related to a decline in domains that are typically affected relatively early in neurodegenerative diseases such as AD (episodic memory [26], working memory [27], and visuospatial abilities [28]) and in cerebrovascular disease (perceptual speed [29]), consistent with our prior publications [30].

**Table 4:** Associations between brain insulin signaling measures and the decline of late-life cognitive function after adjusting for *APOEε4* allele (N=116).

Predictors	Estimate (SE, p-value)					
	Global Cognition	Episodic Memory	Working Memory	Semantic Memory	Perceptual Speed	Visuospatial Abilities
ELISA						
pS^307^IRS1/total IRS1						
Main Effect	-0.071 (0.121,0.560)	-0.149 (0.150,0.322)	-0.110 (0.093,0.238)	0.060 (0.142,0.671)	0.069 (0.122,0.573)	0.021 (0.096,0.827)
Interaction with Time	-0.002 (0.013,0.875)	-0.002 (0.015,0.916)	-0.001 (0.009,0.891)	0.005 (0.015,0.772)	0.001 (0.013,0.917)	-0.002 (0.008,0.800)
pT^308^AKT1/total AKT1						
Main Effect	-0.228 (0.092,0.015)	-0.320 (0.113,0.006)	-0.204 (0.070,0.004)	-0.102 (0.111,0.357)	-0.133 (0.094,0.158)	-0.122 (0.073,0.099)
Interaction with Time	-0.017 (0.010,0.092)	-0.020 (0.012,0.093)	-0.017 (0.007,0.022)	-0.003 (0.012,0.802)	-0.011 (0.010,0.274)	-0.014 (0.006,0.024)
Immunohistochemistry						
pS^616^IRS1 cells/mm^2^						
Main Effect	-0.105 (0.080,0.242)	-0.108 (0.111,0.335)	-0.055 (0.070,0.429)	-0.157 (0.105,0.137)	-0.073 (0.090,0.419)	-0.115 (0.071,0.106)
Interaction with Time	0.001 (0.010,0.908)	-0.003 (0.012,0.810)	0.004 (0.008,0.599)	-0.003 (0.012,0.796)	0.007 (0.010,0.481)	-0.001 (0.007,0.914)

Note: All linear mixed-effects regression models were adjusted for age at death, sex, education, *APOEε4*, and their interaction with time. Data were from 116 participants with at least two longitudinal observations of cognitive function and available information on *APOEε4* status. Bold values denote statistical significance (p<0.05).

Previous research has suggested several potential mechanisms by which brain insulin signaling could be linked to cognitive decline. Some evidence suggests that activation of the AKT1 phosphorylation along the insulin signaling pathway contributes to the development of AD. Specifically, enzyme activities of AKT1 (GSK3α/3β fusion protein phosphorylation by immunoprecipitated AKT1) in temporal cortex were significantly increased in patients with AD, compared with non-disease controls, and the activities were correlated with Braak staging for neurofibrillary changes [10]. In addition, the levels of phosphorylated AKT1 and the phosphorylated downstream effectors including GSK3β were also significantly increased in AD compared with controls [11]. These findings, consistent with ours, might suggest phosphorylation activation of AKT1 and phosphorylation inactivation of GSK3β as a compensatory mechanism to ameliorate AD pathology. Yet, disentangling signaling pathways remains very complicated, and more research with modern tools promises to shed further light on this and related questions.

Another line of research shows that insulin receptors are highly concentrated at the synapses in the brain [31], and insulin signaling plays a crucial part in neurosynaptic functioning [32]. In this regard, insulin facilitates the induction of hippocampal activity-dependent synaptic plasticity (i.e., long-term potentiation or LTP, and long-term depression or LTD), a cellular basis for learning and memory [33], via activation of PI3K-AKT1 signaling pathway [34]. By the same mechanism involving AKT1, insulin induces the inactivation of glycogen synthase kinase-3β (GSK3β) during LTP to prevent synapses from undergoing LTD, permitting the initial consolidation of newly formed memory [35]. Yet another possibility is that insulin in the brain increases neuronal glucose uptake via AKT1-activated glucose transporter type 4 (GLUT4) translocation [36]. Since GLUT4 is preferentially expressed in brain regions important for cognitive function including the basal forebrain, hippocampus, amygdala, cerebral cortex, and cerebellum [37], this process is thought to be particularly important in situations of high cognitive demand, such as during learning [38, 39]. Apart from neurons, insulin signaling, especially along the PI3K-AKT1 pathway, also maintains the physiological functions of other cell types in the brain, such as astrocytes and microglia, which support neuronal survival and normal cognition [40]. Consistent with these data, our findings in this study further highlight the role of insulin signaling in brain aging, by showing that increased AKT1 phosphorylation among community-based older individuals is associated with a faster decline in global cognitive function, and three cognitive domains (episodic memory, working memory, visuospatial abilities). The involvement of these domains implicates multiple brain regions, including the entorhinal cortex as well as the prefrontal cortex [41-43], from where the insulin and related measures were collected. Such cognitive domains are known to be affected relatively early in the development of AD [26-28]. Future research is needed to confirm our findings and investigate whether normalizing brain insulin signaling along the AKT1 pathway can slow down the late-life cognitive decline and the development of AD.

We found that lower IRS1 phosphorylation was associated with a lower level and faster decline in perceptual speed. While phosphorylation of IRS1 at S^307^ has been linked to insulin resistance, whether it is part of a negative or positive feedback loop that controls insulin signaling remains controversial and could be tissue-dependent [13, 44, 45]. Static levels of serine phosphorylation of IRS1 are hard to interpret for their net effect on insulin signaling. We speculate that lower pS^307^IRS1/total IRS1 leads to more brain insulin resistance and vascular disease, which contribute to the decline of perceptual speed [29, 30]. This speculation is supported by a previous study conducted by our group showing that brain insulin resistance is associated with more cerebral infarcts in the postmortem brain[46]. More studies are warranted to elucidate the underlying mechanisms.

In line with our prior published data [17], we did not find any association of pS^616^IRS1-stained cell density assessed using immunohistochemistry with the level or decline of global cognition or any of the five cognitive domains. However, in stratified analyses, we found that in those with diabetes, a higher density of pS^616^IRS1-stained cells was associated with a lower level, but not a faster decline in semantic memory. These findings should be interpreted with caution, since there is well-recognized, considerable variability in immunohistochemical staining and technical challenges in the study of postmortem tissues and with longer duration of freezer storage [47]. More research using independent samples and other complementary approaches is needed to confirm our results.

This study has several limitations. First, the research participants were older volunteers who were relatively healthy, community-dwelling Catholic priests, brothers, and nuns, who agreed to annual evaluations and autopsies at the end of their life. The study design with enrollment of Catholic clergy, introduces selection bias and additional confounding, such that these factors may decrease the generalizability of our findings. On the other hand, the greater homogeneity of environment and lifestyle factors may allow better sensitivity to detect associations with clinical parameters of interest in smaller samples than might otherwise be necessary. Second, we only measured levels of insulin signaling in the prefrontal cortex. Data on other brain regions related to cognitive function such as the hippocampus and posterior parietal cortex were not available. Future research will need to investigate regional differences in brain insulin signaling. Third, other insulin signaling molecules or pathways we did not assess could be also important in the association between brain insulin resistance and cognitive decline and may warrant further investigation. For instance, we did not assess the AKT1 phosphorylation at S473. Yet, we previously did not find an association of insulin-stimulated pS473AKT1 levels with cognitive function proximate to death [17]. Last, the sample size is relatively small. This may lead to missing potentially important associations, as suggested by analyses with *APOEε4*. The small sample also did not allow us to use nonlinear models throughout the analyses, as convergence can be an issue with unimportant predictors. Therefore, we used a naïve assumption of linear decline first and then carefully examined in subsequent analyses, the key findings with nonlinear models. In future studies with larger sample sizes, these complex analyses can be fully employed to understand the trajectories. Despite these limitations, this study has several important strengths. First, we collected longitudinal detailed neuropsychological test data on global cognition and five domain domains, among very well clinically characterized community-dwelling older adults with and without diabetes. The mean duration of annual follow-up was 9.4 years, which allowed us to rigorously model the trajectory of late-life cognitive decline. Second, the brain insulin signaling data were derived from biospecimens with a short postmortem interval, which increases the validity of the brain measurements. Last, we characterized brain insulin signaling using complementary approaches, including both biochemical and immunohistochemical measures.
